# A retrospective review of mortality and complications following oesophagectomy in a large UK teaching hospital

**DOI:** 10.1186/cc13249

**Published:** 2014-03-17

**Authors:** N Pawley, CM Ball, K Wickenden, B Riley, M Clapham, B Eltayeb, A Glossop, A Raithatha

**Affiliations:** 1 Sheffield Teaching Hospital, Sheffield, UK

## Introduction

Just over 1,200 curative oesophagectomies are carried out in the UK annually. Although in-hospital mortality rates have fallen (12 to 13% in 1998 to 2.5% 2013), complication rates remain high [1] with anastomotic failure and respiratory failure common postoperatively [2]. The aim of this retrospective review was to examine the outcomes in patients who underwent oesophagectomies in our unit between January 2010 and October 2012.

## Methods

We examined demographic data, survival (30 day and 1 year) and length of ICU and hospital stay. Case notes were reviewed to identify postoperative complications including anastomotic breakdown, reintubation and respiratory failure. Data were analysed to examine the relationship between the use of postoperative non- invasive ventilation and intraoperative fluid volume and the incidence of postoperative complications.

## Results

Seventy-two patients were identified as having undergone an oesophagectomy between January 2010 and October 2012. Median age was 65 and 82% were male. One patient died within 30 days (1.39%) and nine patients had died by 1 year (12.5%). The median length of ICU and hospital stay was 4 days and 14 days respectively. Six patients had an anastomotic leak (of which two were chyle leaks). Use of non- invasive ventilation (in 23.1% of patients) was not associated with an anastomotic leak (chi-square *P *= 0.53), nor was the amount of fluid given intraoperatively (Mann-Whitney U *P *= 0.410). Six patients had to be reintubated and this was associated with a significantly increased length of both ICU and hospital stay (Mann-Whitney U *P *= 0.01 and 0.03 respectively). Lower P/F ratios were also associated with a significant increase in length of both ICU and hospital stay (*P *= 0.007 and 0.043).

## Conclusion

The overall mortality and morbidity rate was comparable with that seen nationally. Our data suggest that the use of non-invasive ventilation was not associated with anastomotic breakdown. A lower P/F ratio in the postoperative period was associated with prolonged ICU and hospital stay.

## References

[B1] National Oesophago-cancer Audit Annual Report 2013. The NHS Information Centre https://catalogue.ic.nhs.uk/publications/clinical/oesophago-gastric/nati-din-audi-supp-prog-oeso-gast-canc-2013/din-audi- supp-prog-oeso-gast-2013-rep.pdf

[B2] Michelet P Br J Surg 2009 96 54 60 10.1002/bjs.6307 19108006

